# Health-related quality of life outcomes in pediatric patients with cardiac rhythm devices: a cross-sectional study with case-control comparison

**DOI:** 10.1186/s12955-019-1219-5

**Published:** 2019-10-11

**Authors:** Helene Werner, Phaedra Lehmann, Alina Rüegg, Silvia Hilfiker, Karin Steinmann, Christian Balmer

**Affiliations:** 1 Department of Psychosomatics and Psychiatry, University Children’s Hospital, Steinwiesstrasse 75, CH-8032 Zurich, Switzerland; 2 Division of Child and Adolescent Health, Institute of Psychology, University of Zurich, Binzmühlestrasse 14, Box 8, CH-8050 Zürich, Switzerland; 3 Children’s Research Center, University Children’s Hospital, Steinwiesstrasse 75, CH-8032 Zurich, Switzerland; 4 Department of Cardiology, Pediatric Heart Centre, University Children’s Hospital, Steinwiesstrasse 75, CH-8032 Zurich, Switzerland

**Keywords:** Cardiology, Arrhythmia, Children, Adolescents, Adjustment, Adaptation, Predictors

## Abstract

**Background:**

Little is known about health-related quality of life (HRQoL) in pediatric patients with cardiac rhythm devices. This study aims to compare self- and proxy-reported HRQoL in patients with pacemaker (PM) and implantable cardioverter-defibrillator (ICD) to that in sex- and age-matched healthy controls and to examine predictors for generic and disease-specific HRQoL.

**Methods:**

The study included 72 PM and ICD patients (39% females) and 72 sex- and age-matched healthy controls from 3 to 18 years of age. HRQoL data was obtained by the PedsQL 4.0 Generic Core Scales and Pediatric Cardiac Quality of Life Inventory. Medical data was collected retrospectively from medical records.

**Results:**

Patients had significantly lower self- and proxy-reported generic overall HRQoL and lower physical health than healthy controls, and ICD patients also had lower psychosocial health. On multivariate analyses, generic overall HRQoL and physical health was significantly predicted by current cardiac medication (*β* = −.39, *p* = .02 for overall HRQoL, respectively *β* = −.44, *p* = .006 for physical health). Disease-specific overall HRQoL was only marginally predicted by child age, device type, and the presence of a structural congenital heart disease (*p* < .10).

**Conclusions:**

This study shows that PM and ICD patients have lower HRQoL than healthy controls and that patients who need cardiac medication are seen by their parents at great risk for lower generic overall HRQoL. Our study also indicates a trend towards higher risk for low disease-specific HRQoL in younger patients, ICD patients, and patients with a structural congenital heart disease. Special attention should be given to these patients as they may benefit from a timely clinical evaluation in order to provide supportive interventions.

## Background

Pacemakers (PMs) and implantable cardioverter-defibrillators (ICDs) have proven to be life-saving therapeutic options for the treatment of cardiac arrhythmia. Over time, safer implant techniques and smaller devices have been introduced, and today, even very young patients can benefit from a permanent cardiac rhythm device [1, 2]. In pediatric patients, the most common indication for PM implantation is post-operative or congenital advanced atrioventricular block. The implantation of an ICD is indicated for primary or secondary prevention of sudden cardiac death caused by ventricular fibrillation or ventricular tachycardia, associated with congenital heart disease (CHD), genetic arrhythmia syndromes, and cardiomyopathies [2, 3]. Both interventions face various challenges such as arrhythmia-related symptoms, post-operative complications (e.g. infections or pleural effusion), device-related complications (e.g. lead failure or battery depletion), and regular outpatient visits for clinical and device examination. In fact, pediatric patients with PM or ICD deal with life-long dependence on medical assistance and must cope with restrictions in daily activities and reduced life expectancy [4, 5]. This can be very stressful and negatively impact their health-related quality of life (HRQoL).

HRQoL is a multidimensional concept that evaluates the subjective perception of the impact of health status on physical, psychological, and social functioning and well-being [6]. Knowledge of HRQoL is important and can help to identify subjects who are at risk for health problems and need professional assistance. Thus, it can be used to adequately support patients in their adjustment to chronic disease. Previously, few studies have described both generic and disease-specific HRQoL in pediatric patients with PMs, with ICDs, or both [7–9]. The majority of studies that used a generic HRQoL instrument indicated that patients with PMs or ICDs or both have lower HRQoL levels than healthy controls [7–14]. However, medical risk factors for impaired HRQoL have not been studied systematically. One of these studies with PM and ICD patients identified the presence of CHD and ICD as key drivers of lower HRQoL [7]. Two studies found no association between generic HRQoL and cardiac illness severity in ICD patients [8, 12], and one other study found left ventricular ejection fraction lower than 55% and intake of cardiovascular drugs associated with lower generic HRQoL [15].

The aims of this study were threefold. Firstly, we wanted to compare self- and proxy-reported generic HRQoL outcomes in PM and ICD patients to those in sex- and age-matched healthy controls. Lower HRQoL was expected in patients than in healthy controls. Secondly, we wanted to describe the level of self- and proxy-reported disease-specific HRQoL in PM and ICD patients: We expected that ICD patients would show lower HRQoL than PM patients. Thirdly, we aimed to examine associations between patients’ socio-demographic and medical characteristics and proxy-reported generic and disease-specific HRQoL. Based on previous results [7], we expected to identify lower HRQoL in ICD patients and in patients with structural CHD.

## Methods

### Participants

This comparative cross-sectional study includes pediatric cardiac rhythm device patients and sex- and age-matched healthy controls from 3 to 18 years of age. All PM and ICD patients, with or without complaints, scheduled for regular follow-up visits at the cardiology outpatient clinic of the University Children’s Hospital Zurich between September 2015 and September 2016 were asked to participate in the study. Patients with PM or ICD implantation within the last 3 months prior to study were excluded to minimize the influence of acute medical effects on HRQoL. Further exclusion criteria were permanent residency outside Switzerland, lack of German language fluency in patient or caregiver, refusal to sign the informed consent, the presence of Down syndrome, and severe mental retardation. The healthy controls were matched in age and sex and recruited via best friends of the patients (40%), and advertisements placed at the University of Zurich and community day care centers (60%). Interviews and standardized questionnaires ensured that the controls had no chronic disease or cognitive impairments. The study was approved by the Ethical Review Board of the Canton of Zurich, Switzerland, and was performed in full accordance with the Declaration of Helsinki. All parents provided written informed consent after the study procedures and aims had been explained in detail. All pediatric patients provided verbal assent, while adolescents older than 14 years of age also provided written informed consent.

In total, 90 pediatric patients with PM or ICD were eligible for study inclusion. Of these, 72 patients were included (response rate 80%). Eighteen patients did not participate, for the following reasons: lack of time or interest (*n* = 13), lost to follow-up (*n* = 4), and twins (*n* = 1; both twins took part in the study, but only 1 child was randomly selected for inclusion in the analysis, because parent-reported data on HRQoL are correlated in such cases). Study participants did not significantly differ from the 18 non-participants in their sex (χ^2^ = .19, *p* = .79), age at study beginning (U = -.98, *p* = .33), device type (χ^2^ = .02, *p* = .99), age at initial device implantation (U = -.93, *p* = .35), presence of structural CHD (χ^2^ = .18, *p* = .79), or total length of cardiac hospitalization (U = -.80, *p* = .43).

### Measurements

The time since initial device implantation (years) was calculated by the difference between the child’s age at HRQoL assessment and age at initial device implantation. ICD patients were grouped by whether they had experienced an aborted cardiac arrest or life-threatening arrhythmia (secondary prevention) or not (primary prevention) [16]. The severity of the disease in ICD patients was defined by using the ICD severity index [12]. Patients with structural CHDs were categorized by the complexity of the surgical repair into univentricular (i.e. Fontan type) and biventricular physiology. Previous open-heart surgery was dichotomized into yes or no. The number of device-related post-initial-implant surgeries was categorized into none, one, or more than one. The length of each hospital stay related to cardiac disease was summed into the total length of cardiac hospitalization (days). Patients’ current cardiac medication was assessed by the intake of heart failure and/or antiarrhythmic medication, dichotomized into yes or no. In addition, the presence of any other, non-cardiac chronic disease (e.g. neurological diseases with recurrent seizures) was noted.

Generic HRQoL was assessed using the validated and authorized German version of the Pediatric Quality of Life (PedsQL) 4.0 Generic Core Scales [17]. It is a widely used 23-item measure for assessing HRQoL during the past month by proxy-reports in the age range of 2 to 18 years and by child self-report in the age range of 5 to 18 years. This instrument encompasses the following 4 subscales: physical functioning (8 items), social functioning (5 items), emotional functioning (5 items), and school functioning (5 items). The physical health summary score is defined by the physical functioning subscale. The psychosocial health summary score is computed as the sum of the items over the number of items answered in the emotional, social and school functioning subscales. In addition, a total score (generic overall HRQoL) can be computed as the sum of all the items divided by the number of items answered on all subscales. Items are reverse scored and linearly transformed to a 0–100 scale, with higher scores indicating better HRQoL.

Disease-specific HRQoL was assessed using an authorized German version of the standardized Pediatric Cardiac Quality of Life Inventory (PCQLI), which was supplemented by a preschool instrument [18, 19]. Thus, we used the PCQLI consisting of two self-report versions (for ages 8–12 and 13–18) and three proxy-report versions (for ages 3–7, 8–12, and 13–18). Both self- and proxy-reported versions include 27 items (ages 8–12) and 38 items (ages 13–18) comprising three subscales: ‘impact of disease’, ‘psychosocial impact’, and ‘emotional environment’. A total score for disease-specific overall HRQoL is calculated by the sum of the ‘impact of disease’ and ‘psychosocial impact’ subscales. The proxy-report version for preschool children (ages 3–8) includes 52 items. A total score, computed by the sum of all items, defines the overall disease-specific HRQoL. All scales were linearly transformed into a 0–100 scale, with higher scores indicating better HRQoL. Cut-off scores for very low, low, and normal disease-specific HRQoL total scores are available calculated from a German sample of 546 children with heart diseases [20].

The occurrence of major life events in the family during the 12 months prior to assessment was assessed by parental report, using a list of the following 12 life events: birth of a child, divorce, marriage, person moving into the household, significant change in family income, indebtedness, relocation, job change of either parent, unemployment of either parent, serious illness or accident of a family member, death of family member or a close friend, and child’s change of school [21]. A life event score was computed by summing the number of life events (range 0–12).

Child nationality and socioeconomic status (SES) were assessed by a short parent-reported socio-demographic questionnaire. SES was assessed by maternal education and paternal occupation on a scale from 2 to 12, with 2 being the lowest and 12 the highest SES score. Three social classes were defined: lower (SES 2 to 5), middle (SES 6 to 9), and upper class (SES 10 to 12). This measure has proven to be a valid indicator of SES in previous studies involving the Swiss population [22].

### Study procedure

After informed consent had been provided, HRQoL data was obtained using standardized questionnaires. To assess proxy-reports of child HRQoL from 3 to 18 years, questionnaires were mailed to participating parents, who completed them at home. Because valid and reliable self-reported HRQoL data can be assessed from 7 to 8 years of age [23], no self-reports were assessed for patients younger than 7.5 years. For older patients, self-reported HRQoL data was obtained in face-to-face interviews that strictly followed the procedure in the questionnaire. To ensure that the patients could express their own views openly, they were interviewed separately from their parents. The interviews were conducted by the first, second, or third author, all of whom had been trained. Self- and proxy-reported HRQoL data was assessed within 2 weeks. Patients’ medical data was collected retrospectively from medical records.

### Statistical analyses

Data were analyzed using the SPSS statistical package, release 22.0 for Windows (SPSS Inc., Chicago, IL, USA). All statistical tests were two-sided with a predefined significance level of *p* < .05. Chi-square tests and Mann-Whitney U-tests were used, as appropriate, to compare child sex, age at study beginning, device type, age at device implantation, the presence of structural CHD and the total length of cardiac hospitalization between study participants and non-participants. The differences in generic HRQoL between patient sample and control group and between self- and proxy-reports were analyzed by testing first the interaction between study group (patient vs. control) and report (self vs. proxy), and then the differences between patient and control group on one side and between self- and proxy-reports on the other side (see caption of Table 2). Associations between medical variables and generic and disease-specific HRQoL scores (self- and proxy-reports) were measured by non-parametric Kendall’s rank correlation coefficients. Because some medical variables were continuous while others were categorical or dichotomous, we decided to use this coefficient in all cases. Four separate multiple linear regression models with identical variables were used to predict HRQoL (with the generic overall HRQoL score, the physical health and psychosocial health summary score, and the disease-specific overall HRQoL score as dependent variables). Selection of predictors was based on a priori hypotheses and on the statistical significance of bivariate correlations with the total score. Eight predictors were entered: child sex, age at assessment (years), time since initial device implantation (years), device type, structural CHD, current cardiac medication, total length of cardiac hospitalization (days), and other non-cardiac chronic disease. The absence of multi-collinearity was confirmed by examining the correlation matrix (correlations > .80) and the variance inflation factor [24].

## Results

Patient socio-demographic and medical characteristics for the total sample and the two device types are summarized in Table 1. The majority of the patients (82%) had a PM device, while 13 patients (18%) had an ICD. Patients with an ICD were significantly older at assessment and initial device implantation than patients with PM. Four of the 59 PM patients (7%) and 5 of the 13 ICD patients (39%) had experienced resuscitation before implantation. Overall, 40 of the 72 PM and ICD patients (56%) had a CHD, and 33 of these (83%) had also undergone open-heart surgery. The time since last device-related surgery was significantly shorter in ICD patients than in PM patients, andICD patients needed more antiarrhythmic medication than PM patients. In total, drug therapy for heart failure or arrhythmia was present in 23 of 72 patients (32.9%), with 3 patients (4.2%) who needed both medications, 14 patients (19.4%) antiarrhythmic medication only, and 3 patients (4.2%) heart failure medication only. An additional non-cardiac chronic disease was present in 13 of 72 patients (18%): attention deficit hyperactivity disorder (*n* = 3), hearing disorder (*n* = 1), congenital hand or arm malformation (*n* = 2), diabetes mellitus (*n* = 1), neurological disease (*n* = 5), and diseases of the endocrine system (*n* = 1).

**Table 1:** Patient socio-demographic and medical characteristics by cardiac rhythm device type

	Total sample (*n* = 72)	PM (*n* = 59)	ICD (*n* = 13)	Comparison: PM vs. ICD group *p* value^†^
Female sex, n (%)	28 (39)	25 (42)	3 (23)	.23
Age at assessment (years), mean (SD), range	11.3 (4.6), 3.2–18.0	10.5 (4.4), 3.2–17.9	14.9 (3.2), 7.6–18.0	**<.001**
Swiss nationality, n (%)	59 (82)	47 (80)	12 (92)	.44
Socio-economic status, n (%)				.23
Lower class	3 (4)	3 (6)	0	
Middle class	49 (68)	38 (64)	11 (84)	
Upper class	17 (24)	16 (27)	1 (8)	
Unknown	3 (4)	2 (3)	1 (8)	
Number of previous life events, mean (SD), range	2.2 (1.9), 0–7	2.3 (2.0), 0–7	1.7 (1.3), 0–4	.44
Age at initial device implant (years), mean (SD), range	4.7 (4.4), 0–15.3	3.5 (3.5), 0–12.6	9.8 (4.0), 2.9–15.3	**<.001**
Newborn (age ≤ 28 days), n (%)	7 (10)	7 (12)	0	
Infant/toddler (29 days to 3 years), n (%)	23 (32)	22 (37)	1 (8)	
Preschool (3 to 6 years), n (%)	18 (25)	17 (29)	1 (8)	
School-age (6 to 13 years), n (%)	21 (29)	13 (22)	8 (62)	
Adolescent (age > 13 years), n (%)	3 (4)	0	3 (23)	
Time since initial device implant (years), mean (SD), range	6.7 (4.5), 0.4–17.9	7.1 (4.6), 0.4–17.9	5.1 (3.9), 0.5–12.7	.23
Device system, n (%)				
Single-chamber	9 (13)	4 (7)	5 (39)	
Dual-chamber	63 (87)	55 (93)	8 (61)	**.002**
Location of electrode, n (%)				
Epicardial	69 (96)	59 (100)	10 (77)	
Endocardial	3 (4)	0	3 (23)	NA
Electrophysiological disease, n (%)				
Postoperative heart block	20 (28)	20 (34)		
Congenital heart block	25 (35)	25 (42)		
Sinoatrial node disease	14 (19)	14 (24)		
Ventricular tachycardia or fibrillation^a^	13 (18)		13 (100)	NA
ICD indication, n (%)				
Primary prevention	NA	NA	7 (54)	
Secondary prevention	NA	NA	6 (46)	NA
ICD Severity Index, mean (SD), range	NA	NA	8.0 (3.8), 3–16	NA
Structural congenital heart disease, n (%)				
Biventricular physiology	34 (47)	26 (44)	8 (61)	
Univentricular physiology	6 (8)	6 (10)	0	
No structural congenital heart disease	32 (45)	27 (46)	5 (39)	.34
Previous open heart surgery, n (%)	33 (46)	30 (51)	3 (23)	.07
Number of device-related post-initial-implant surgeries, n (%)				
None	38 (53)	33 (56)	5 (39)	
One	21 (29)	15 (25)	6 (46)	
More than one	13 (18)	11 (19)	2 (15)	.25
Time since last device-related surgery (years), mean (SD), range	3.5 (2.6), 0.3–12.7	3.9 (2.6), 0.3–12.7	1.8 (1.5), 0.3–5.4	**.004**
Total length of cardiac hospitalization (days), mean (SD), range	52.4 (32.5), 2–266	57.0 (62.4), 2–266	31.5 (33.0), 3–133	.26
Current cardiac medication				
Antiarrhythmic, n (%)	17 (24)	7 (12)	10 (77)	**<.001**
Heart failure, n (%)	6 (8)	5 (9)	1 (8)	.93
Other non-cardiac chronic disease, n (%)	13 (18)	9 (15)	4 (31)	.19

*NA* Not applicable, *PM* Pacemaker, *ICD* Implantable cardioverter-defibrillator

^†^ Chi square tests or Mann-Whitney-U tests were performed

^a^ Patients with documented ventricular tachycardia or fibrillation or with a significant risk of ventricular tachycardia or fibrillation

Significant values are indicated in bold

The sex- and age-matched healthy controls did not differ significantly from the patient sample in Swiss nationality (patient group: 82%; control group: 83%). In contrast, controls had significantly higher SES: 33 of the 72 controls (46%) belonged to the upper SES class, but only 17 of the 72 patients (24%) did. Furthermore, parents of patients reported having experienced more previous life events than parents of controls (*p* < .001; patient group: M = 2.2, SD = 1.9, range = 0–7; control group: M = 0.8, SD = 1.0, range = 0–4). In both samples, death of a family member or a close friend (patient sample: 39%, control group: 18%) and a job change of either parent (patient sample: 34%, control group: 21%) were the most frequently reported life events.

Mean values of self- and proxy-reported generic HRQoL scores for the patient sample and control group are presented in Table 2 separately for the PM and ICD groups. None of the interactions between study group (patient vs. control group) and report (self vs. proxy) was significant. This means that average HRQoL differences between patients and controls are the same for self- and proxy-reported HRQoL data. Our analyses showed that parents of PM and ICD patients reported significantly lower overall HRQoL and lower physical health summary scores than did parents of healthy controls. In addition, parents of ICD patients reported lower psychosocial health summary scores. No significant differences were found between self- and proxy-reported generic HRQoL scores.

**Table 2:** Sample means for generic health-related quality of life in pediatric patients with PM/ICD and their controls: comparison between patients and control group and between self- and proxy report

	Parent proxy-report form					Child self-report form					Interaction: Study group (patient/control) vs. report (self/proxy)	Differences Patient sample vs. control group (all control – patient differences)		Differences Self- vs. proxy report (all self – proxy differences)	
	Patient sample			Control group		Patient sample			Control group						
	Device type	n	M (SD)	n	M (SD)	Device type	n	M (SD)	n	M (SD)	*p*- value^†^	M (SD)	*p*- value	M (SD)	*p*- value
PedsQL scales															
Total Score	PM	57	83.0 (14.5)	59	89.9 (8.1)	PM	39	82.1 (11.6)	42	86.7 (12.2)	.34	7.1 (17.8)	**.007**	−1.3 (9.8)	.21
Physical Health Summary	PM	57	86.4 (16.5)	59	94.6 (8.5)	PM	39	84.0 (13.9)	42	87.6 (14.2)	.24	8.7 (18.4)	**≤.001**	−2.8 (12.6)	.06
Psychosocial Health Summary	PM	57	79.6 (14.3)	59	85.1 (9.6)	PM	39	80.3 (11.6)	42	85.8 (11.9)	.85	5.4 (19.0)	.057	0.1 (12.5)	.78
Emotional Functioning	PM	57	73.2 (17.2)	59	75.6 (15.1)	PM	39	76.5 (16.6)	42	82.5 (12.8)	.26	2.2 (26.1)	.69	1.5 (16.3)	.93
Social Functioning	PM	57	85.9 (19.2)	59	91.0 (11.9)	PM	39	84.6 (13.6)	42	90.0 (15.5)	.84	5.0 (23.4)	.16	−1.1 (13.9)	.94
School Functioning	PM	55	80.1 (17.1)	56	88.7 (12.2)	PM	39	79.6 (13.4)	42	84.8 (14.1)	.43	9.4 (20.5)	**.002**	0.0 (18.9)	.41
PedsQL scales															
Total Score	ICD	13	75.8 (16.0)	13	92.2 (7.0)	ICD	12	77.7 (14.9)	13	91.0 (7.2)	.16	16.4 (15.1)	**.009**	2.1 (17.5)	.41
Physical Health Summary	ICD	13	76.0 (17.4)	13	95.9 (5.9)	ICD	12	76.0 (19.8)	13	90.1 (9.4)	.17	20.0 (16.5)	**.005**	−0.3 (22.8)	.53
Psychosocial Health Summary	ICD	13	75.6 (16.8)	13	88.6 (11.3)	ICD	12	79.4 (12.2)	13	91.8 (5.7)	.75	12.9 (17.8)	**.02**	4.4 (15.0)	.33
Emotional Functioning	ICD	13	72.3 (23.3)	13	84.6 (14.1)	ICD	12	79.2 (14.4)	13	89.2 (9.8)	.62	12.3 (21.4)	.06	7.1 (17.9)	.21
Social Functioning	ICD	13	80.4 (19.1)	13	93.5 (11.3)	ICD	12	82.5 (13.7)	13	95.4 (8.3)	.79	13.1 (21.5)	.06	1.7 (22.4)	.39
School Functioning	ICD	13	74.2 (18.0)	13	87.7 (13.0)	ICD	12	76.7 (15.1)	13	90.8 (7.9)	.88	13.5 (21.4)	.06	4.6 (18.6)	.73

Higher scores indicate better health-related quality of life

*PedsQL* Pediatric quality of life inventory, *PM* Pacemaker, *ICD* Implantable cardioverter defibrillator

^†^ Calculation for the interaction: Let Y_PPr_, Y_CPr_, Y_PS_, Y_CS_ define HRQoL of the patients and controls for self- and proxy reports, respectively. Then the difference (Y_PPr_-Y_CPr_) – (Y_PS_-Y_CS_) was calculated and tested against 0 by Wilcoxon test

Significant values are indicated in bold

**Fig. 1:**
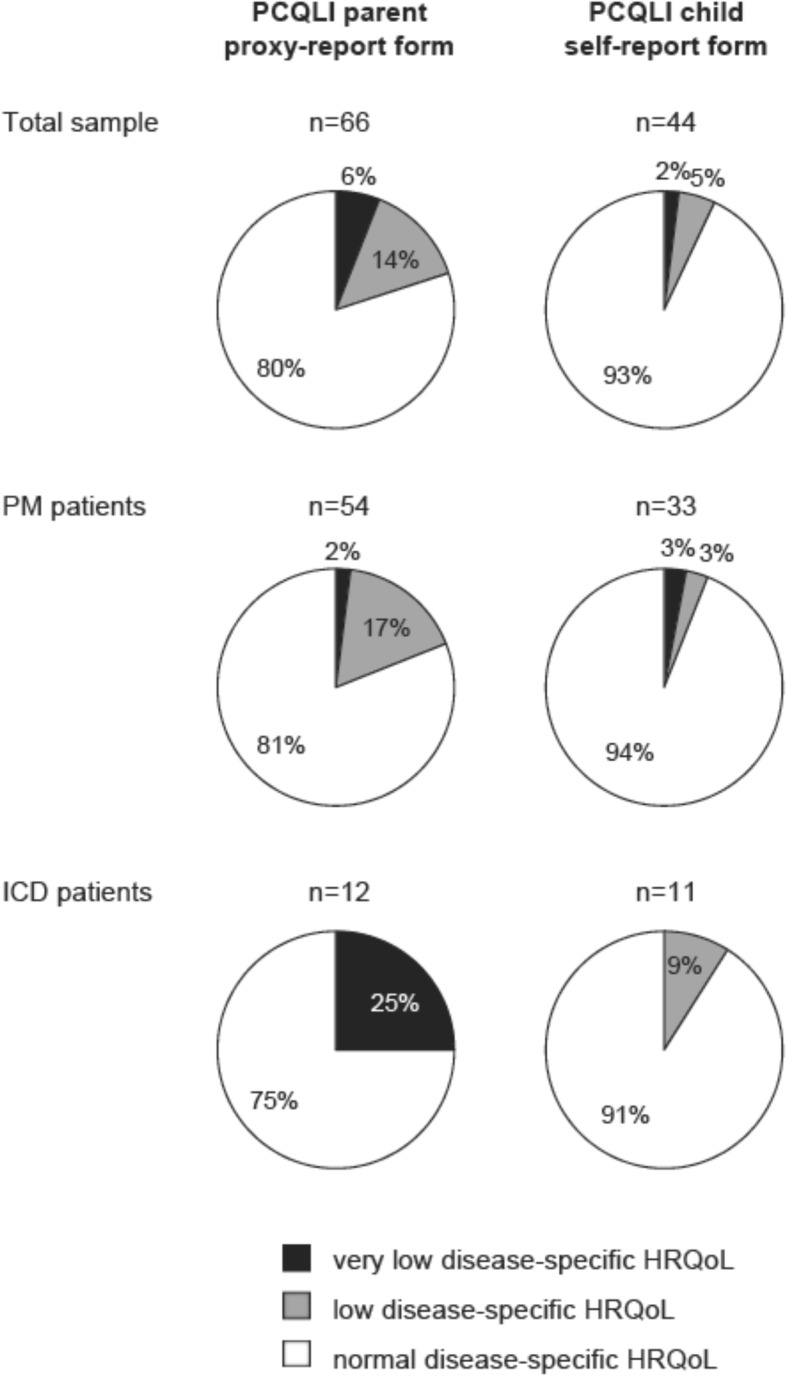
Number of pacemaker (PM) and implantable cardioverter-defibrillator (ICD) patients with very low, low, and normal disease-specific health-related quality of life (HRQoL) assessed by the Pediatric Cardiac Quality of Life Inventory (PCQLI)

The number of patients with very low, low, and normal disease-specific HRQoL (proxy- and self-reported data) are indicated in Fig. 1. The majority of the patients have normal proxy- and self-reported disease-specific HRQoL. Chi-square tests revealed that ICD patients more frequently had very low proxy-reported disease-specific HRQoL than did PM patients (χ^2^ = 10.7, *p* = .005). No such difference was found in the self-reported data between PM and ICD patients (χ^2^ = 1.01, *p* = .60). Among ICD patients, 25% of the parents reported very low disease-specific HRQoL for their child, while no patient reported very low and 9% reported low disease-specific HRQoL. Thus, significant differences between self- and proxy-reported disease-specific HRQoL scores were found in all subscales and the total score (*p* = .002, *d* = −.47).

Associations between generic or disease-specific HRQoL scores and socio-demographic and medical characteristics are presented in Table 3. Kendall correlation coefficients showed that generic HRQoL scores were not significantly associated with patients’ socio-demographic characteristics, while it was associated with current cardiac medication. Disease-specific HRQoL scores were significantly associated with child age at assessment, the presence of a structural CHD and a longer total length of cardiac hospitalization.

**Table 3:** Inter-correlations between total generic and disease-specific HRQoL scores (proxy-report form) and child socio-demographic and medical characteristics

	All patients (*n* = 66 to 72)												
	1	2	3	4	5	6	7	8	9	10	11	12	13
1. Total generic HRQoL	–												
2. Total disease-specific HRQoL	.48***	–											
3. Female sex	−.03	−.06	–										
4. Age at assessment (years)	.02	.24**	−.07	–									
5. Swiss nationality	.10	.12	.00	.18	–								
6. Socio-economic status	.06	.07	.19	−.17	.21	–							
7. Number of previous life events	−.13	−.08	.00	.11	−.17	.03	–						
8. Time since initial device implant (years)	−.01	.15	−.02	.38***	.02	−.10	.31***	–					
9. Device type (PM or ICD)	.16	.16	.15	−.32***	−.13	.13	.08	.12	–				
10. Structural congenital heart disease	−.15	−.35***	−.09	−.16	.02	−.13	−.09	.05	−.06	–			
11. Current cardiac medication	−.33***	−.31**	−.18	.11	.13	−.07	−.20	−.12	−.60***	.24*	–		
12. Total length of cardiac hospitalization (days)	−.13	−.21*	−.08	−.15	−.03	−.07	−.08	.14	.11	.57***	.21*	–	
13. Previous open heart surgery	−.03	−.17	−.05	−.15	−.00	−.09	−.12	.09	.21	.82***	.11	.59***	–
14. Other non-cardiac chronic disease	−.19	−.07	−.08	.18	.13	.19	.20	.17	−.16	−.02	.11	−.00	−.14

Kendall correlation coefficients are presented

Device type coded as 0 = ICD, 1 = PM; Structural congenital heart disease coded as 0 = no, 1 = yes; Current cardiac medication coded as 0 = no, 1 = yes; Previous open heart surgery coded as 0 = no, 1 = yes; Other chronic disease coded as 0 = no, 1 = yes (any other chronic disease than heart disease)

*HRQoL* Health-related quality of life

* *p* ≤ .05, ** *p* ≤ .01, *** *p* ≤ .001

Table 4 summarizes the results of four separate multiple regression analyses predicting proxy-reported generic and disease-specific HRQoL (generic overall HRQoL score, physical health and psychosocial health summary, and disease-specific overall HRQoL). In regression models predicting generic HRQoL, child sex and age were not significant predictors. Among the medical variables, current cardiac medication was a significant predictor for overall HRQoL and the physical health summary score, while no significant association was found with the psychosocial health summary score. The need for cardiac medication was associated with lower generic overall HRQoL and physical health. In addition, the presence of another non-cardiac chronic disease was associated with lower physical health. Disease-specific overall HRQoL was only marginally associated with child age at assessment, device type, and structural CHD (*p* < .10). Younger patients, patients with ICD, and those with a structural CHD had lower disease-specific HRQoL than older patients, patients with PM, and those with no CHD.

**Table 4:** Summary of multiple linear regression analyses predicting proxy-reported generic and disease-specific HRQoL of patients with PM/ICD

Generic HRQoL	Generic overall HRQoL (*n* = 70)					Physical Health Summary					Psychosocial Health Summary				
Predictor variable	B	SEB	β	t	*p*	B	SEB	β	t	*p*	B	SEB	β	t	*p*
Female sex	−2.98	3.49	−.10	−.86	.40	−4.54	3.91	−.13	− 1.16	.25	−1.43	3.59	−.05	−.40	.69
Age at assessment (years)	.47	.55	.14	.85	.39	.33	.62	.09	.53	.60	.61	.57	.19	1.08	.29
Time since initial device implant (years)	−.66	.53	−.19	−1.25	.22	−.64	.59	−.17	− 1.08	.28	−.67	.54	−.20	−1.24	.22
Device type (PM or ICD)	2.12	6.35	.06	.33	.74	2.01	7.11	.05	.28	.78	2.23	6.53	.06	.34	.73
Structural congenital heart disease	2.69	4.10	.09	.66	.51	3.46	4.59	.10	.75	.45	1.93	4.21	.07	.46	.65
Current cardiac medication	−12.73	5.14	−.39	−2.48	**.02**	−16.45	5.75	−.44	−2.86	**.006**	−9.02	5.28	−.28	−1.71	.09
Total length of cardiac hospitalization (days)	−.05	.04	−.18	−1.27	.21	−.04	.04	−.13	−.93	.36	−.06	.04	−.22	−1.45	.15
Other non-cardiac chronic disease	−8.69	4.50	−.22	−1.93	.06	−10.20	5.04	−.23	−2.03	**.05**	−7.19	4.62	−.19	−1.56	.13
	*F* = 2.78, *p* = .01, *R*^2^ = .27, *R*^2^adjusted = .17					*F* = 3.11, *p* = .005, *R*^2^ = .29, *R*^2^adjusted = .20					*F* = 1.93, *p* = .07, *R*^2^ = .20, *R*^2^adjusted = .10				
Disease-specific HRQoL	Disease-specific overall HRQoL (*n* = 66)														
Predictor variable	B	SEB	β	t	*p*										
Female sex	−4.14	3.54	−.13	−1.17	.25										
Age at assessment (years)	1.06	.55	.31	1.91	.06										
Time since initial device implant (years)	.06	.53	.02	.10	.92										
Device type (PM or ICD)	12.03	6.43	.29	1.87	.07										
Structural congenital heart disease	−7.72	4.21	−.24	−1.83	.07										
Current cardiac medication	−4.50	5.10	−.13	−.88	.38										
Total length of cardiac hospitalization (days)	−.04	.04	−.14	−.98	.33										
Other non-cardiac chronic disease	−5.88	4.50	−.14	−1.31	.20										
	*F* = 4.17, *p* ≤ .001, *R*^2^ = .37, *R*^2^adjusted = .28														

Device type coded as 0 = ICD, 1 = PM; Structural congenital heart disease coded as 0 = no, 1 = yes; Current cardiac medication coded as 0 = no, 1 = yes; Other chronic disease coded as 0 = no, 1 = yes (any other chronic disease than heart disease)

*B* Regression coefficient (unstandardized), *SEB* Standard error of the regression coefficient, *β* Standardized regression coefficient

Significant values are indicated in bold

## Discussion

This study shows that pediatric PM and ICD patients have lower generic HRQoL than age- and sex-matched healthy controls, and that patients who need cardiac medication are seen as being at great risk for reduced generic HRQoL. The study also shows that the majority of the patients have normal proxy- and self-reported disease-specific HRQoL. Further, younger patients, patients with ICDs, and those with a structural CHD may have a higher risk for lower proxy-reported disease-specific HRQoL.

In line with our hypothesis and with previous studies with PM patients, ICD patients, or both [7, 8, 10–13], pediatric PM and ICD patients showed lower self- and proxy-reported overall HRQoL and lower physical health than sex- and age-matched healthy controls. Lower psychosocial health was only found in ICD patients. In our study, healthy controls had higher SES and had experienced fewer previous life events than the patients. Consequently, we examined whether these variables might influence the HRQoL differences between patients and controls. A multiple linear regression with generic overall HRQoL differences as dependent variable showed no significant effect of SES or of number of previous life events on HRQoL differences between patients and controls (data not shown). A multitude of risk factors might influence the significant HRQoL differences between patients and controls (e.g. mental disorder of a parent, marital disharmony, lack of social support [25]). However, PM and ICD patients have to cope with at least one more life event than do healthy controls. The presence of a chronic disease with associated challenges such as arrhythmia-related symptoms, post-operative complications, device-related complications, and regular outpatient visits for clinical and device examination, which might be experienced as a threat exceeding the resources of the patients and their families [26] might be reflected in reduced HRQoL. Our study showed that parents of 20% of the patients and 7% of the patients themselves reported very low or low disease-specific HRQoL. Thus, some PM and ICD patients are at great risk for low HRQoL. Our data shows that parents and patients reported about the same level of generic HRQoL, while parents reported lower disease-specific HRQoL than the patients themselves. Thus, we may assume that the parents’ subjective experience of the child’s disease may especially bias their disease-specific proxy-reports.

In multivariate analyses, generic and disease-specific HRQoL were not predicted by the same variables. This probably reflects the scope of the questionnaires used. We decided to use the PedsQL 4.0 Generic Core scales [17] and the PCQLI [18, 19]. While the PedsQL assesses the full range of health conditions (physical, social, emotional, and school functioning) and can be used to report HRQoL independently of the actual health state of the individual [27], the PCQLI refers to the unique challenges of a heart disease [27] and, thus, may more comprehensively report the challenges faced by PM and ICD patients [28]. This is also shown by the higher percentage of total variance explained by the predictors selected for disease-specific HRQoL. Our study showed that the need for antiarrhythmic or heart failure medication was associated with lower generic overall HRQoL and physical health. This finding is in line with the only previous study which analyzed the association between medication and HRQoL exclusively in PM patients [15]. Thus, pediatric PM and ICD patients who take medication are seen as being at risk for lower generic HRQoL by their parents. Three hypotheses are close: First, it could be assumed that these patients have greater disease-related problems than patients without medication. Second, it might be possible that these patients already had a lower HRQoL before the onset of their arrhythmia and that patients with low HRQoL are more likely to be prescribed medication. Third, it might be that the intake of medication reminds the patients and their parents of the disease, which can be reflected in lower HRQoL scores. However, in the context of this study, we were not able to prove these hypotheses and whether medication is an intermediating factor or not. Future studies might address this. Disease-specific HRQoL was only marginally predicted by child age, device type, and structural CHD. Younger patients, patients with ICDs, and those with CHD tended to have lower disease-specific HRQoL than older patients, patients with PM, and those without CHD. This result is in line with a previous study’s finding that patients with ICD and a structural CHD have lower HRQoL [7].

The strengths of the current study include the use of standardized and well-validated HRQoL instruments and the presentation of data on both generic and disease-specific HRQoL, which are seen as essential complements to each other [23] and which allow the influence of medical risk factors on each to be compared. Nevertheless, several limitations merit note. First, even though we achieved a response rate of 80% and our analyses revealed no significant differences between study participants and non-participants in sex, age at study beginning, device type, age at initial device implantation, presence of structural CHD, or the total length of cardiac hospitalization, we cannot exclude the possibility that the patients who participated in our study differed with respect to other variables (e.g. HRQoL) from those who did not. In addition, the possibility that some cross-contamination occurred between patients and controls cannot be excluded, since 40% of controls were recruited via patients’ best friends. Second, the cross-sectional design of our study prevents us from drawing any conclusion about causal relations, and we are not able to describe the course of HRQoL over time. Third, our study included a rather small number of ICD patients, making it difficult to compare both device groups with respect to socio-demographic and medical characteristics. In terms of a post-hoc power analysis (α = .05, two-tailed) using G*power software [29], our sample size provided sufficient power to detect moderate effect sizes within the multiple regression analysis predicting proxy-reported generic and disease-specific HRQoL. However, we were not able to predict self-reported generic and disease-specific HRQoL. Fourth, our study shows that socio-demographic and medical factors explain only a relatively small proportion of the patients’ HRQoL outcome variance. This seems to imply that other factors not assessed in the current study influence the patients’ HRQoL. In fact, we were unable to provide data on patients’ social support or parental well-being or to define its proportion of variance explaining either generic or disease-specific HRQoL.

## Conclusions

Our study shows that PM and ICD patients have lower generic overall HRQoL compared to healthy controls and that patients who need cardiac medication are at great risk for lower generic HRQoL. Furthermore, our study indicated a trend towards higher risk for low disease-specific HRQoL in younger patients, ICD patients, and patients with a structural CHD. Special attention should be given to these patients. They may benefit from a timely clinical evaluation of their HRQoL in order to provide supportive interventions.

## Data Availability

The datasets used and analyzed during the current study are available from the corresponding author on reasonable request.

## Abbreviations

HRQoL: Health-related quality of life
ICD: Implantable cardioverter-defibrillators
PCQLI: Pediatric Cardiac Quality of Life Inventory
PM: Pacemakers
SES: SocioEconomic Status
